# Design, Characterization, and Release Kinetics of a Hybrid Hydrogel Drug Delivery System for Sustained Hormone Therapy

**DOI:** 10.3390/polym17080999

**Published:** 2025-04-08

**Authors:** Mohammed E. Ali Mohsin, Akhtar Jahan Siddiqa, Suleiman Mousa, Nilesh Kumar Shrivastava

**Affiliations:** 1 Department of Chemical Engineering, College of Engineering, King Faisal University, P.O. Box 400, Al Ahsa 31982, Saudi Arabia; 2 Materials Science Centre, Indian Institute of Technology, Kharagpur 721302, West Bengal, India

**Keywords:** hybrid-hydrogel system, letrozole, sustained drug release, release kinetics, hormone therapy

## Abstract

This study presents a hybrid hydrogel system designed for the targeted delivery of letrozole, a key therapeutic agent in breast cancer treatment. Letrozole-loaded poly(lactic-co-glycolic acid) (PLGA) microparticles were embedded within a poly(2-hydroxyethyl methacrylate) (pHEMA) matrix coated onto acrylamide-grafted low-density polyethylene (AAm-g-LDPE), yielding a mechanically stable system with tunable drug release. Field emission scanning electron microscopy (FE-SEM) and confocal microscopy confirmed uniform microparticle distribution. In vitro release studies in simulated uterine fluid (SUF) at 37 °C demonstrated a sustained release profile over 32 days, with a reduced initial burst effect (~15% lower than conventional PLGA systems). The system’s release kinetics followed the Higuchi model (*R*^2^ = 0.803–0.996), indicating Fickian diffusion. This hybrid hydrogel offers enhanced drug stability, reduced dosing frequency, and potential for personalized hormone therapy, improving patient compliance, particularly for individuals with physical or cognitive impairments.

## 1. Introduction

In controlled drug delivery, it is essential to attain specific release patterns for therapeutic substances from polymeric setups like hydrogels and micro- or nanoparticles. Compared to immediate-release versions, controlled-release forms offer several advantages [1,2,3,4]. Therefore, achieving a sustained and controlled release profile is crucial for drug efficacy, particularly for drugs with a narrow therapeutic index [5,6,7]. Recent advancements in drug delivery systems aim to achieve near-zero-order release, ensuring consistent therapeutic levels over time. However, many current systems exhibit burst release or incomplete drug retention, necessitating novel approaches like hybrid hydrogels for better control. This study examines the effectiveness of a polymeric heterogeneous setup designed to release therapeutic agents at a specific rate and quantity. This system comprises a continuous hydrophilic polymer matrix containing micro-sized sub-matrices encapsulating letrozole. The release of the enclosed compounds can be controlled by utilizing both the primary polymer matrix and the sub-matrices.

The preparation techniques for this hybrid system primarily involve chemical reactions to create a heterogeneous structure by blending the sub-matrices into a monomer solution, which then undergoes polymerization to form the heterogeneous matrix [8,9]. Typically, the process of fabricating a polymeric composite device via solvent removal entails initially integrating the drug into polymeric sub-matrices. These sub-matrices are then suspended in a monomer solution, wherein they are insoluble, resulting in a viscous solution. The system is formed after solvent removal [10,11,12,13,14]. Several researchers have synthesized and characterized various delivery systems based on this principle. Marra et al. [15] developed a tissue-engineering scaffold integrated with drug-loaded microparticles. Both the scaffold and microparticles were crafted from PLGA with different ratios of PLA and PGA [16]. Additionally, PVA was employed to coat PLGA (50/50) microparticles to shield them from organic solvents when integrated into PLGA (65/35) scaffolds, and the release of bovine albumin was assessed. PLA microspheres were embedded in either a polyurethane or polystyrene matrix to achieve prolonged release of heparin, with slower release observed in polystyrene rather than polyurethane as the matrix polymer [17]. Brannon-Peppas et al. [18] devised a composite system comprising PLGA particles dispersed in silicone matrices to examine the release of 3-estradiol. They noted that the release rate of 3-estradiol fell between the release rates observed from microparticles and silicone films alone, respectively. However, the incorporation of PLGA microparticles into the silicone matrix involved curing the mixture at 100 °C, exceeding the *T_g_* of PLGA (~40 °C). Consequently, the high temperature caused deformation and loss of integrity in the PLGA microparticles, obscuring the structure and morphology of the PLGA–silicone composite. Nonetheless, these composite systems primarily focus on integrating microparticles into hydrophobic polymer matrices.

Some researchers have directed their attention towards incorporating biodegradable micro- or nanoparticles into hydrogel matrices to explore sustained or controlled drug release [11,12,13,14,19,20]. Hydrogels, due to their high-water content and soft, rubbery elasticity, bear a resemblance to living tissue in terms of physical properties. Consequently, a hybrid system wherein drug-loaded nanoparticles are embedded within a hydrogel matrix holds promise for controlled therapeutic agent release. Such systems would exhibit different release kinetics compared to typical hydrogel matrices or nanoparticles.

Since the PLGA (75:25) copolymer is relatively hydrophobic, a heterogeneous system was designed to take advantage of the solubility differences between HEMA and PLGA. Unlike previous hydrogel systems for hormone therapy, which focus on peptide hormones or microgel encapsulation [21,22], our design integrates PLGA microparticles into a pHEMA matrix on an AAm-g-LDPE backbone. Initially, PLGA microparticles containing letrozole were prepared and then dispersed in HEMA to achieve a uniform mixture. This mixture was subsequently polymerized onto AAm–g–LDPE to fabricate the LET–PLGA-pHEMA/AAm–g–LDPE (heterogeneous system) release platform.

## 2. Experimental Procedure

### 2.1. Materials

Poly(lactic-co-glycolic acid) (PLGA) with a 65:35 ratio of lactide and glycolide (Mw ≈ 40,000–75,400 Da, purity ≥ 98%) was procured from Sigma-Aldrich (St. Louis, MO, USA). 2-Hydroxyethyl methacrylate (HEMA, density 1.07 g/cm^3^ at 20 °C, purity ≥ 97%) was obtained from Merck (Darmstadt, Germany). Potassium persulfate (KPS, purity ≥ 99%) was procured from Merck Specialities Pvt. Ltd., (Mumbai, India), and used as a redox initiator for the polymerization reaction. Laboratory reagent grade polyvinyl alcohol PVA (Mw ≈ 125,000 Da, density at 20 °C = 1.31 g/cm^3^, purity ≥ 98%) was sourced from SD Fine Chemicals (Mumbai, India). *N*,*N*,*N*′,*N*′-Tetramethylethylenediamine (TEMED, purity ≥ 99%) was procured from LOBA Chemie Pvt. Ltd. (Mumbai, India) and was used as an accelerator for the polymerization reaction. *N*,*N*-methylenebisacrylamide (BIS, purity ≥ 99%) was obtained from SISCO Research Laboratories Pvt. Ltd. (Mumbai, India). Letrozole (4,4′-((1H-1,2,4-triazol-1-yl) methylene) dibenzonitrile, purity ≥ 98%) and dichloromethane were purchased from Sigma-Aldrich (St. Louis, MO, USA). Deionized water was used to prepare the SUF.

### 2.2. Preparation of Letrozole-Encapsulated PLGA-pHEMA/AAm–g–LDPE Drug Release System

**Figure 1:**
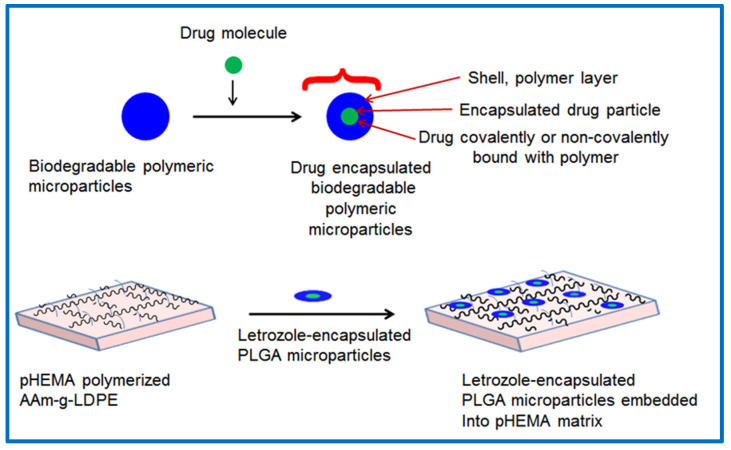
Schematic presentations of PLGA microparticles embedding in a pHEMA-coated layer on an AAm–g–LDPE drug release system.

The preparation and characterization of letrozole-encapsulated PLGA microparticles (F1, F2, F3, and F4) were detailed in a previous study [23]. These formulations were selected for the preparation of the hybrid drug release system investigated in this study. Letrozole-encapsulated PLGA microparticles with different formulations containing 0.5%, 1%, and 2% (*w*/*w*) were dispersed in diluted HEMA monomer. The remainder of the coating polymerization process proceeded as follows: AAm–g–LDPE films were immersed in LET–PLGA–HEMA solution under nitrogen purging to remove dissolved oxygen. The initiator, KPS, was introduced into the reaction chamber and homogenized for a few minutes. Subsequently, BIS (cross-linker) was added, and the reaction mixture was homogenized. A schematic of this process is depicted in Figure 1. Following this, 10 µL of TEMED (accelerator) was added to the reaction mixture. After approximately 10 min, the monomer started developing viscosity, after which the reaction mixture was degassed before polymerization and sealed with Parafilm^®^ M. The reaction systems were left to polymerize at 22 °C for 24 h. Subsequently, the prepared systems were immersed in a mixture of deionized water and ethanol and washed three times to remove any unreacted monomers and chemicals. Finally, the samples were dried to a constant weight at room temperature under vacuum.

## 3. Results and Discussion

### 3.1. Analysis of Particle Distribution Within the pHEMA Matrix of the Prepared Release System

**Figure 2:**
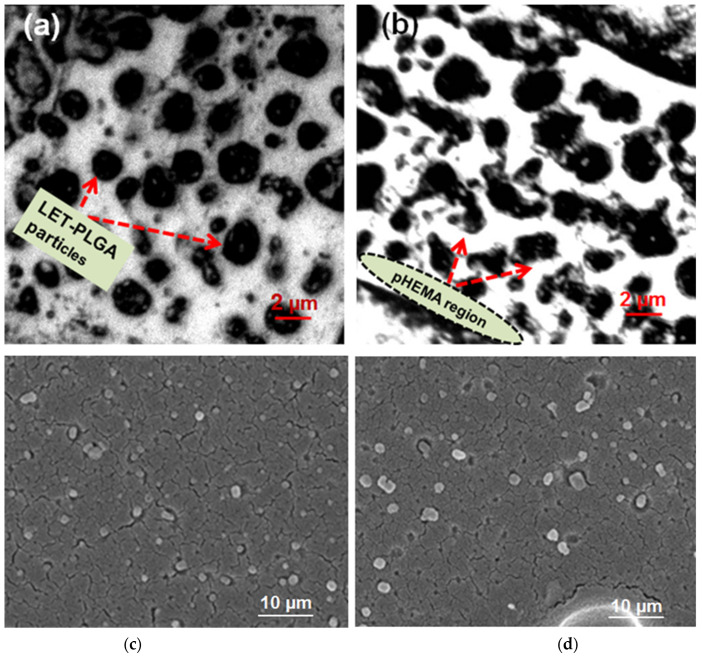
(**a**,**b**) FE-SEM micrographs of PLGA microparticles-encapsulated letrozole embedded within the pHEMA matrix and (**c**,**d**) images of confocal laser scanning microscopy of LET-PLGA particle distribution in pHEMA matrix. (Magnification = 40×).

The distribution of letrozole–PLGA microparticles within the pHEMA matrix (coated on the AAm–g–LDPE) was examined using field emission scanning electron microscopy (FE-SEM). The samples were mounted on stubs and coated with a 10 nm layer of gold to prevent sample charging. The resulting micrographs are presented in Figure 2, revealing well-dispersed particles scattered throughout the pHEMA matrix while maintaining their original shape and morphology. As FE-SEM and SEM primarily detect surface morphology rather than internal distribution within the matrix, confocal laser scanning microscopy (CLSM, IX81) was employed to further analyze the uniformity of PLGA microparticle distribution within the pHEMA matrix.

The prepared samples were cut transversely to expose the embedded microparticles within the pHEMA matrix and then vacuum-dried. Images were captured by scanning the laser beam through the probe [24]. Confocal images depicted two distinct regions: one consisting of spherical dark particles and the other displaying a transparent region. The transparent region represents the polymerized pHEMA matrix, while the dark region consists mainly of letrozole-loaded PLGA particles. This confirms the uniform distribution of microparticles within the pHEMA matrix. Furthermore, the number of particles increased with greater scanning depth. Additionally, Figure 2c demonstrates spherical particle shapes, while Figure 2d exhibits some particles with slight deformation, likely caused by stirring during particle mixing in the HEMA monomer, generating stress between the particles and pHEMA matrix. This stress may have influenced the particles to adopt a slightly elliptical shape. Nevertheless, the particles are uniformly dispersed throughout the matrix. The size and size distribution of letrozole-loaded PLGA microparticles were characterized using dynamic light scattering (DLS) as reported in our previous study [23], with average diameters ranging from 5 to 15 µm (polydispersity index, PDI = 0.25 ± 0.03). Following incorporation into the pHEMA matrix, the hybrid hydrogel system’s overall thickness was measured via cross-sectional FE-SEM, revealing a uniform coating of 50–70 µm. The microparticle distribution within the matrix remained consistent, with no significant aggregation observed, as confirmed by confocal microscopy (Figure 2).

### 3.2. In-Vitro Release Study

In vitro release assessments were conducted using the prepared letrozole-encapsulated PLGA–pHEMA/AAm–g–LDPE in simulated uterine fluid (SUF, pH 7.6) to evaluate the release pattern of letrozole from the heterogeneous system. The system was submerged in 10 mL SUF within a 50 mL beaker and placed in an incubator at 37 °C with a shaking speed of 40 rpm. At predefined intervals, 1 mL aliquots were withdrawn, and an equal volume of fresh SUF was replenished. The drug concentration in the aliquots was measured using a NanoVue spectrophotometer at 238 nm. Each release study was conducted in triplicate.

**Figure 3:**
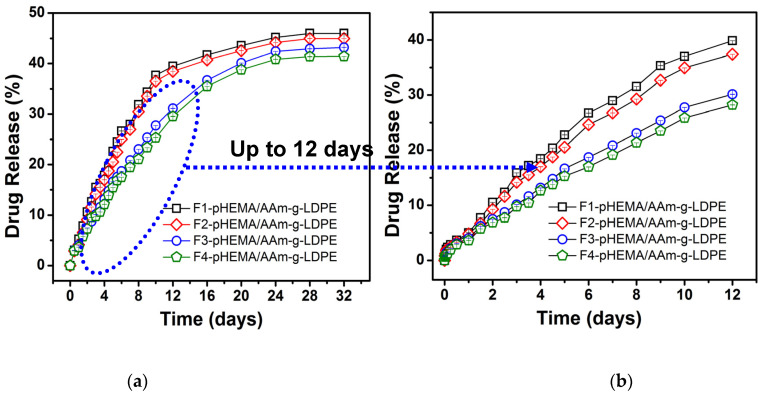
In vitro release of letrozole from letrozole–PLGA–pHEMA/AAm–g–LDPE in SUF at 37 °C (**a**) up to 32 days and (**b**) first 12 days. Results are mean ± SD (n = 3).

The drug release profiles are depicted in Figure 3a. All systems exhibited a biphasic release pattern throughout the 32-day release study. Initially, a low release profile was observed (4 ± 0.3% in 24 h), followed by a lag phase until day 12, succeeded by a second phase of relatively slow and sustained release. The incorporation of formulations F1 and F2 into the pHEMA matrix resulted in 34.5 ± 1.2% and 34 ± 1.1% drug release over the initial 12 days, respectively, while F3 and F4 showed 31 ± 1.0% and 30 ± 1.0% drug release, indicating minimal burst release. The reduced initial burst release could be attributed to the incorporation of PLGA particles into the pHEMA matrix, creating an additional barrier for drug diffusion into the medium. Furthermore, the deposition of pHEMA onto the particle surface contributed to the decrease in burst release. Consequently, a linear release pattern with a low slope was observed during the initial phase (as shown in Figure 3b for the initial 12 days). Following this, the release rate decelerated, maintaining a constant (or sustained) level until the end of the study period (32 days). The delayed and reduced drug release can be attributed to the prolonged hydration of PLGA, which delays the hydrolysis of PLGA and subsequently reduces the amount of drug released. This stage of release may involve drug diffusion through the pores.

In the case of PLGA nanoparticle-encapsulated letrozole embedded within the pHEMA matrix (F3 and F4), the drug release exhibited a low and constant profile. Approximately 41 ± 1.5% and 43 ± 1.6% of the drug was released within the 32-day study period for F3 and F4, respectively. This slow release may be attributed to factors such as particle size or the presence of a thick layer around the drug in the particles, which prolongs the core layer hydrolysis process. Thicker layers lead to slower hydrolysis, increased diffusion distance for drug molecules within the particles, and consequently slower release. Additionally, the physicochemical properties of the polymer (PLGA 75:25) and letrozole, both being hydrophobic and insoluble in water, contribute to the slowed release rate. The sustained release over 32 days would gradually erode the particle layers in the aqueous environment, suggesting that the release mechanism involves a combination of diffusion, particle swelling, and polymer erosion. Compared to conventional PLGA microparticle systems, our hybrid hydrogel showed a more consistent release profile, reducing initial burst effects by approximately 15 ± 0.9% (Figure 3). These findings align with recent reports by Liu B et al. [24], demonstrating the advantages of hydrogel matrices in drug stabilization. The encapsulation efficiency (EE) of letrozole in PLGA microparticles (F1–F4) ranged from 78.5 ± 2.1% to 85.3 ± 1.8%, as determined in our prior work [23]. For the hybrid system, EE was slightly lower (75.2 ± 2.3% for F4), likely due to minor drug loss during pHEMA polymerization.

### 3.3. Release Kinetics Study

**Figure 4:**
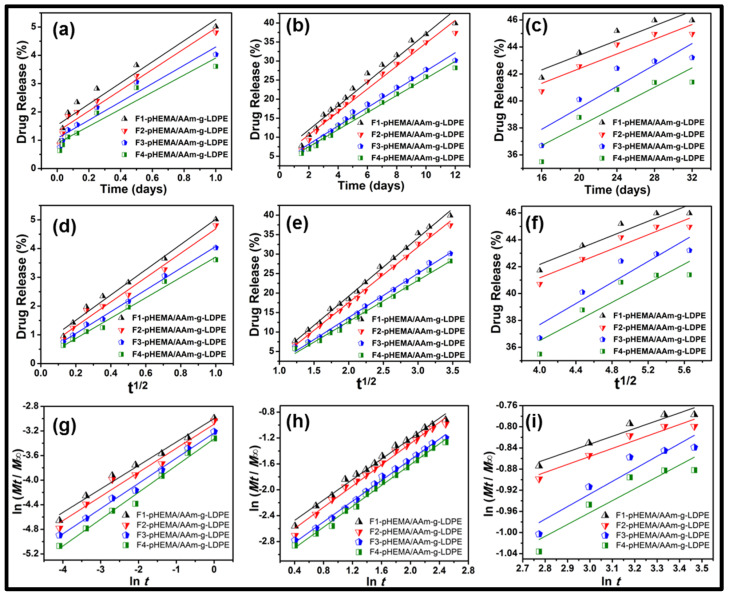
In vitro release kinetics in three stages (**a**) *t*_0_*–t*_1_ day (**b**) *t*_1_*–t*_12_ day and (**c**) *t*_12_*–t*_32_ day after fitting in zero-order model (**d**) *t*_0_*–t*_1_ day, (**e**) *t*_1_*–t*_12_ day, and (**f**) *t*_12_*–t*_32_ day after fitting in Higuchi model (**g**) *t*_0_*–t*_1_ day, (**h**) *t*_1_*–t*_12_ day, and (**i**) *t*_12_*–t*_32_ day after fitting in Korsmeyer–Peppas model of embedded letrozole–PLGA microparticles within pHEMA matrix.

To analyze the release kinetics of letrozole from the system, the release data were fitted to kinetic models as outlined below.

(1) $$\mathrm{Zero}\text{-}\mathrm{order}\;\mathrm{model}\text{:}\;\;\;M_{t}=M_{0}+K_{0}t$$

(2) $$\mathrm{Higuchi}\;\mathrm{model}\text{:}\;\;\;M_{t}=M_{0}+K_{H}t^{1/2}$$

(3) $$\mathrm{Korsmeyer}–\mathrm{Peppas}\;\mathrm{model}\text{:}\;\;\;M_{t}=M_{0}+K_{k}t^{n}\;$$

where *M_t_* represents the amount of drug dissolved in time *t*, *M*_0_ denotes the initial drug amount, *K*_0_ stands for the zero-order release constant, *K_H_* represents the Higuchi rate constant, *K_K_* is a release constant, and *n* is the release exponent characterizing the mechanism of drug release. Release data were fitted to standard models (Zero-order, Higuchi, Korsmeyer–Peppas), with *R*^2^ values confirming a diffusion-controlled mechanism. The Higuchi model showed the best fit, indicating drug release followed Fickian diffusion principles. Consequently, the fitted line is illustrated in Figure 4. The calculated values of the correlation coefficient (*R*^2^), *k*, and *n* for each model at different release stages (I: *t*_0_*–t*_1_, II: *t*_1_*–t*_12_, and III: *t*_12_*–t*_32_ days) are presented in Table 1a–c.

Based on the curve fitting, the linear regression lines of the Higuchi and Korsmeyer–Peppas models provide the equations that best fit the data, with correlation coefficient (*R*^2^) values and line slopes close to constants. The Higuchi model plot displayed the highest linearity, suggesting that drug release from the matrix follows a process dependent on the square root of time, consistent with Fickian diffusion.

The initial phase (Stage I) demonstrated a slow-release profile, which can be attributed to the presence of PLGA particles within the pHEMA matrix, forming an additional diffusion barrier. The Higuchi model exhibited a high correlation (*R*^2^ > 0.98), indicating a diffusion-controlled mechanism during this phase. Comparable behavior has been reported in previous studies involving polymeric hydrogel-based drug delivery systems, where diffusion-controlled release was dominant in the early stages due to polymer swelling and limited water penetration [11].

During Stage II, a more pronounced release was observed, correlating well with both zero-order and Higuchi models, suggesting that drug diffusion and polymer relaxation played a key role. The transition from an initial lag phase to a sustained release phase can be explained by the hydration and gradual erosion of PLGA, which modulated drug diffusion into the surrounding medium. This behavior is consistent with previous findings where hybrid hydrogel systems with embedded nanoparticles exhibited delayed release owing to polymer degradation kinetics [25].

The final phase (Stage III) exhibited a slow and sustained release pattern, best described by the Korsmeyer–Peppas model with release exponent values (n < 0.5), indicating Fickian diffusion as the dominant mechanism. This suggests that, at later stages, drug release was primarily driven by diffusion rather than matrix erosion. Such diffusion-controlled kinetics have been widely observed in PLGA-based hydrogel matrices, where the hydrophobicity of the polymer and the molecular interactions within the system regulate the release profile [26].

Overall, these findings confirm that the hybrid hydrogel system provides a controlled drug release profile, reducing burst release and extending the duration of drug availability in a predictable manner. The incorporation of PLGA within the hydrogel structure enhances the system’s ability to modulate drug diffusion, aligning well with previous studies on hybrid polymeric carriers for sustained drug delivery [27].

The correlation coefficients (R²) obtained with the zero-order model ranged from 0.752 to 0.987 (refer to Table 1a). This model describes drug release from microparticles with low drug solubility and osmotic systems where drug release is directly proportional to time. For the Higuchi model, correlation coefficients (R²) ranged from 0.803 to 0.996. The consistently high correlation coefficients indicate consistent release of letrozole from microparticles after an initial burst effect. This model describes drug release from insoluble matrices through a diffusion process following Fick’s law and a square root of time dependence. In the Korsmeyer–Peppas model, the correlation coefficient (R²) values ranged from 0.837 to 0.996 (as listed in Table 1c). The n values for the Korsmeyer–Peppas equation in both phases were found to be 0.4 ± 0.02 and 0.82 ± 0.04, indicating Fickian and non-Fickian diffusion or an anomalous release mechanism (0.5<n<1), respectively. Anomalous transport results from a combination of Fickian diffusion through hydrated layers of the matrix and relaxation or erosion of polymer chains.

Letrozole release from microparticles occurs through multiple mechanisms, including PLGA hydrolysis, surface and bulk erosion, disintegration, diffusion, and desorption. In this experiment, drug release from the PLGA matrix primarily involves diffusion. In later phases, both drug diffusion and polymer matrix degradation are involved. The acidic monomers and oligomers catalyze further polymer degradation. While bulk-eroding polymers like PLGA 65:35 typically exhibit an initial burst release followed by controlled release, no initial burst effect was observed with all four formulations, indicating homogeneous drug encapsulation in the PLGA matrix. The release pattern can be attributed to the dissolution of the poorly soluble drug from the polymer–drug matrix, involving both polymer erosion and drug solubility into the surrounding media before diffusion through the dialysis membrane.

These findings reinforce the suitability of hydrogel-based matrices for controlled drug delivery applications by minimizing burst release and ensuring prolonged drug availability over extended periods. Future studies could explore optimization strategies such as crosslinking density and polymer composition variations to fine-tune release kinetics.

### 3.4. Comparative Studies on In Vitro Release

A comparative study of letrozole–pHEMA/AAm–g–LDPE, letrozole–PLGA microparticles, and embedded letrozole–PLGA microparticles within the pHEMA matrix was conducted.

**Figure 5:**
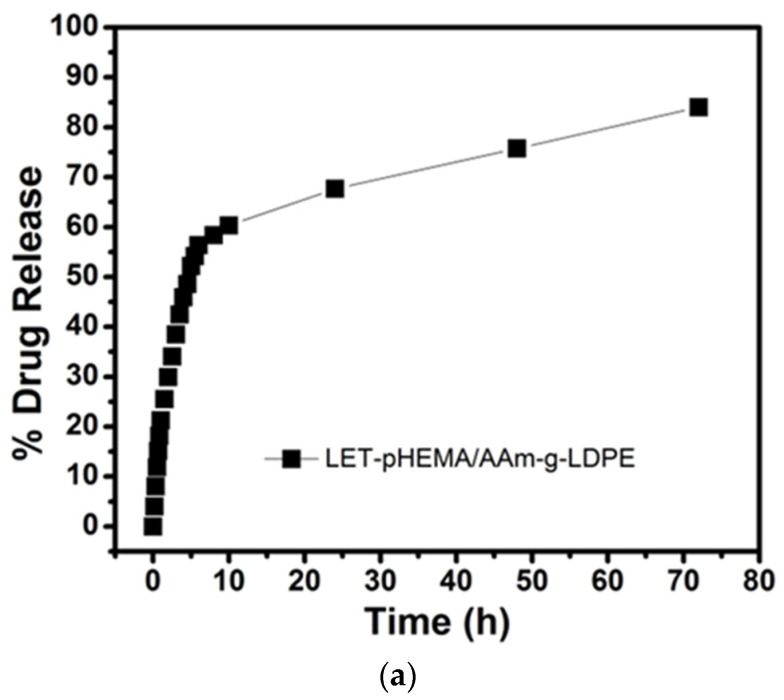

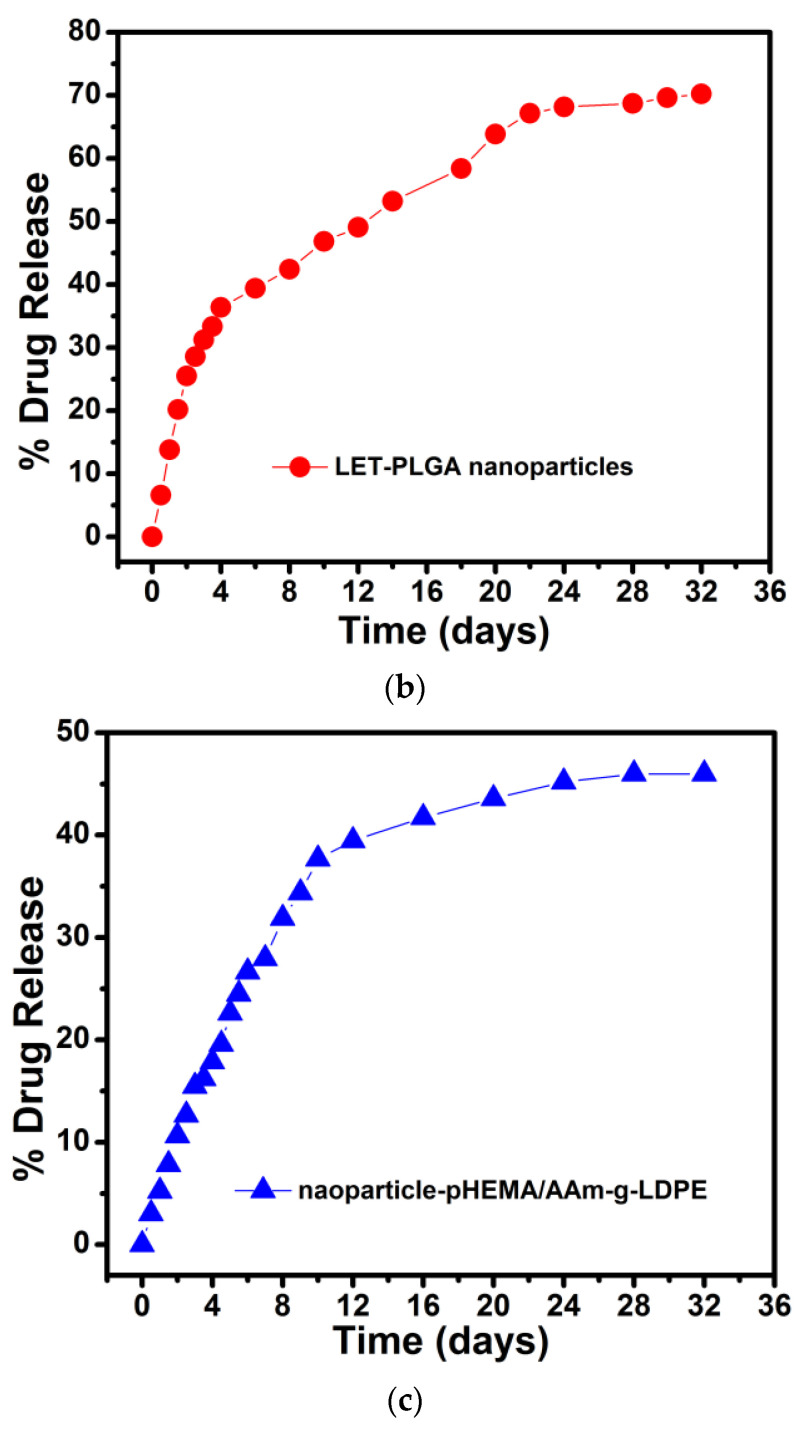
Comparison in drug release between (**a**) letrozole–pHEMA/AAm-g-LDPE, (**b**) letrozole–PLGA microparticles, and (**c**) F4-pHEMA/AAm-g-LDPE.

In the comparative analysis, the in vitro release pattern of letrozole from the pHEMA-coated layer (without microparticles) system, as depicted in Figure 5a, exhibited approximately 48% initial drug release within the first 12 h, followed by a high percentage of drug release over the subsequent 72 h. A sharp increase in the plot indicates a substantial release of letrozole during the early stages of dissolution studies. This phenomenon is attributed to the release of letrozole dispersed near the polymer matrix, accompanied by the swelling of the pHEMA matrix, which facilitates easy diffusion of the drug molecule. Consequently, approximately 89% of the drug was released within 72 h, suggesting that close to 100% of the drug may be released within 5 to 7 days. In the comparative study, the release profile of letrozole from the microparticles alone exhibited a slower release with a lower initial burst, as illustrated in Figure 5b. During the initial burst phase, approximately 33% of the cumulative amount of letrozole was released within the first 12 days. Subsequently, the release profile followed a consistently slow pattern, resulting in the release of approximately 73% of the cumulative amount of letrozole within 32 days.

**Figure 6:**
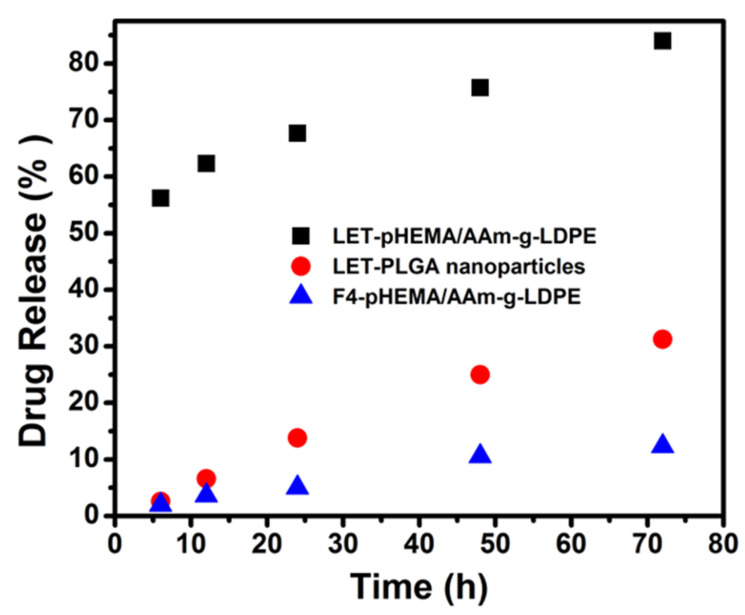
Comparison of drug release behavior over 72 h for (■) LET–pHEMA/AAm–g–LDPE, (●) LET–PLGA nanoparticles, and (▲) F4–pHEMA/AAm–g–LDPE.

The release profile of microparticles embedded into the pHEMA matrix (heterogeneous system) demonstrates nearly constant release, indicating a reduction in the initial release rate (see Figure 5c). This observation may be attributed to the distribution of particles near the surface and core of the matrix, significantly increasing the lag time of PLGA particle hydrolysis. The presence of microparticles in the matrix reduces water absorption capability, consequently decreasing the amount of drug released [28]. Conversely, letrozole–PLGA microparticles alone exhibited approximately 20% more drug release, suggesting the system can sustain drug release for up to 32 days. For comparison, the drug release data from samples of letrozole–pHEMA/AAm–g–LDPE, letrozole–PLGA microparticles, and embedded letrozole–PLGA microparticles within the pHEMA matrix are plotted in Figure 6. The letrozole–pHEMA/AAm–g–LDPE system displayed high drug release over 72 h, with a sharp rise and high slope indicating significant release during the early stages of dissolution studies. This release behavior is attributed to the porous structure of pHEMA, which reaches equilibrium swelling within 52 h, facilitating easy diffusion of letrozole from the matrix.

Release of letrozole from the microparticles alone showed low initial burst release (approximately 25–30%), followed by a slower release rate up to 30 days. Conversely, letrozole–pHEMA/AAm-g-LDPE exhibited a high burst release (64–73%) and a sharp increase within 72 h. In the case of embedded letrozole–PLGA microparticles within the pHEMA matrix, the release system exhibited a very slow but constant release with a lower slope value, indicating that the system can control drug release for an extended period. This suggests that the system can suppress the initial burst release of encapsulated letrozole compared to PLGA microparticles. Additionally, the swollen layer could prevent the penetration of release media into the channels and pores of the PLGA matrix. The presence of a coated cross-linked pHEMA hydrogel layer profoundly affects the release profile of PLGA nanoparticles. The extremely slow-release profile cannot be attributed to drug diffusion through the pHEMA matrix, as drug release from the pHEMA matrix was observed within 72 h. This behavior may result from ester bond cleavage, established as a prevalent mechanism for PLGA nanoparticle degradation and consequent drug release. Compared to an earlier study [29], which highlights hydrogel versatility, our system uniquely combines hydrophobic PLGA microparticles with a hydrophilic pHEMA matrix, achieving a biphasic release with minimal burst and prolonged delivery, outperforming standalone PLGA or pHEMA systems by 20% in sustained release duration.

## 4. Conclusions

This study developed a hybrid hydrogel system embedding letrozole-loaded PLGA microparticles within a pHEMA matrix on AAm-g-LDPE, achieving a sustained 32-day release profile with a ~15% reduction in initial burst compared to conventional PLGA systems. FE-SEM and confocal microscopy confirmed uniform microparticle distribution, while kinetic analysis (Higuchi model, *R*^2^ = 0.803–0.996) indicated diffusion-controlled release. This system addresses the challenge of frequent dosing in hormone therapy, offering stable drug levels and improved patient compliance, particularly for individuals with physical or cognitive impairments. Future studies could explore optimization strategies such as crosslinking density and polymer composition variations to fine-tune release kinetics.

## Figures and Tables

**Table 1:** (a) Release rate constant and correlation coefficients for I stage (t_0_–t_1_ day) of different kinetics models. (b) Release rate constant and correlation coefficients for II stage (t_0_–t_1_ day) of different kinetics models. (c) Release rate constant and correlation coefficients for III stage (t_12_–t_32_ day) of different kinetics models.

Formulation	Zero Order		Higuchi		Korsmeyer−Peppas		
	*R* ^2^	*K*_0_	*R* ^2^	*K_H_*	*R* ^2^	*K_m_*	*n*
(a)							
F1	0.908	1.532 ± 0.045	0.986	0.626 ± 0.032	0.983	−2.299 ± 0.058	0.38 ± 0.02
F2	0.940	1.303 ± 0.038	0.978	0.448 ± 0.025	0.971	−3.089 ± 0.063	0.39 ± 0.02
F3	0.931	1.065 ± 0.041	0.996	0.291 ± 0.027	0.993	−3.233 ± 0.059	0.41 ± 0.03
F4	0.916	0.892 ± 0.036	0.987	0.165 ± 0.019	0.985	−3.327 ± 0.061	0.43 ± 0.03
(b)							
F1	0.972	5.641 ± 0.122	0.995	−10.736 ± 0.287	0.989	−2.789 ± 0.089	0.78 ± 0.04
F2	0.976	4.633 ± 0.118	0.996	−10.935 ± 0.294	0.989	−2.926 ± 0.091	0.82 ± 0.04
F3	0.986	3.381 ± 0.102	0.994	−8.907 ± 0.276	0.996	−3.125 ± 0.087	0.80 ± 0.03
F4	0.987	3.033 ± 0.098	0.993	−8.405 ± 0.264	0.994	−3.221 ± 0.085	0.80 ± 0.03
(c)							
F1	0.849	37.938 ± 1.245	0.888	31.457 ± 1.137	0.916	−1.275 ± 0.057	0.14 ± 0.02
F2	0.849	36.938 ± 1.221	0.888	30.457 ± 1.112	0.916	−1.308 ± 0.059	0.15 ± 0.02
F3	0.786	31.532 ± 1.089	0.833	21.998 ± 0.987	0.863	−1.641 ± 0.062	0.23 ± 0.03
F4	0.752	30.935 ± 1.065	0.803	22.269 ± 0.954	0.837	−1.636 ± 0.061	0.22 ± 0.03

## Data Availability

The original contributions presented in this study are included in the article. Further inquiries can be directed to the corresponding author.
